# Dynamic all-optical drug screening on cardiac voltage-gated ion channels

**DOI:** 10.1038/s41598-018-19412-z

**Published:** 2018-01-18

**Authors:** Jonas Streit, Sonja Kleinlogel

**Affiliations:** Institute of Physiology, University of Bern, Bühlplatz 5, 3012 Bern, Switzerland

## Abstract

Voltage-gated ion channels (VGCs) are prime targets for the pharmaceutical industry, but drug profiling on VGCs is challenging, since drug interactions are confined to specific conformational channel states mediated by changes in transmembrane potential. Here we combined various optogenetic tools to develop dynamic, high-throughput drug profiling assays with defined light-step protocols to interrogate VGC states on a millisecond timescale. We show that such light-induced electrophysiology (LiEp) yields high-quality pharmacological data with exceptional screening windows for drugs acting on the major cardiac VGCs, including hNa_v_1.5, hK_v_1.5 and hERG. LiEp-based screening remained robust when using a variety of optogenetic actuators (ChR2, ChR2(H134R), CatCh, ChR2-EYFP-βArchT) and different types of organic (RH421, Di-4-ANBDQPQ, BeRST1) or genetic voltage sensors (QuasAr1). The tractability of LiEp allows a versatile and precise alternative to state-of-the-art VGC drug screening platforms such as automated electrophysiology or FLIPR readers.

## Introduction

Voltage-gated ion channels (VGCs) are frequently implicated in neural and cardiovascular disorders. This makes them prime drug targets for the pharmaceutical industry but concomitantly also unwanted off-targets with potentially lethal cardiac side-effects^1,2^. Many promising agents either failed clinical trials or were withdrawn from the market after initial admission due to non-specific effects on cardiac VGCs, in some cases due to fatal polymorphic ventricular tachycardia^3,4^. Indeed, less than 0.05% of all compounds that entered preclinical testing become marketed drugs, with cardiac safety and pro-arrhythmic risk being a leading cause of discontinuation^5,6^. Consequently, new drugs acting on VGCs must follow strict safety guidelines to exclude potential pro-arrhythmic activity, summarized in the recently elaborated Comprehensive *in vitro* Proarrhythmia Assay (CiPA)^7^.

VGCs are challenging targets for drug screening since drug interaction is often confined to specific conformational channel states mediated by transient changes in the transmembrane potential. For this reason, drug screening on VGCs is typically performed by voltage-clamp (VC) enabling precise voltage-step protocols^8^. However, the inherent technical nature of automated patch-clamp limits throughput and increases costs^9^. Accordingly, high-throughput optical *in vitro* assays were developed^10–12^. However, they typically manipulate the transmembrane voltage or channel gating by non-physiological means, *i.e*. using artificial extracellular ion compositions or pharmacological agonists. These crude methods cannot control VGCs dynamically and poorly reflect physiological channel gating. An all-optical platform with millisecond bi-directional optogenetic transmembrane voltage control (de- and hyperpolarization) and concurrent optical readout of VGC activity will resolve the above issues and enable powerful, high-throughput and affordable drug profiling, not only in cell lines as shown here, but potentially also in whole organs or animals.

The here introduced light-induced electrophysiology (LiEp) platform, as we named it, employs optogenetic “tandem” proteins^13^ that enable contactless bi-directional control of the transmembrane potential by light. They combine the blue-light actuated cation channel Channelrhodopsin-2 (ChR2)^14^ with the yellow-light actuated ion pump ArchT^15^ as cell “depolarizer” and “hyperpolarizer”, respectively, in a single protein. For proof of concept we tested LiEp-based drug screening on the cardiac VGCs hK _v_1.5, hNa_v_1.5 and hERG, three renowned drug targets. The hERG potassium channel drives one of the main repolarizing currents in the cardiac action potential and has been the primary off-target of new drugs acting on VGCs^16,17^. The human voltage-gated sodium channel hNa_v_1.5, solely responsible for the initial upstroke of the cardiac action potential^18^, was also included in the CiPA safety screening workflow as a potentially fatal off-target. hNa_v_1.5 is, however, also an important target for antiarrhythmic agents^19^ as is hK _v_1.5, which mediates the ultra-rapid activating repolarizing cardiac delayed rectifier current. Since hK_v_1.5 is specifically expressed in the atria^20^, drugs acting on hK_v_1.5 are devoid of the risk to induce ventricular arrhythmia^21^.

In this study, we demonstrate how LiEp reliably reports dose-dependent drug interactions for a palette of compounds known to act on hK _v_1.5, hNa_v_1.5 and hERG channels. We show that the LiEp platform has a comparable sensitivity, precision and reproducibility to voltage clamp (VC) recordings conducted in parallel.

## Results

### LiEp on hNa_v_1.5 channels

**Figure 1:**
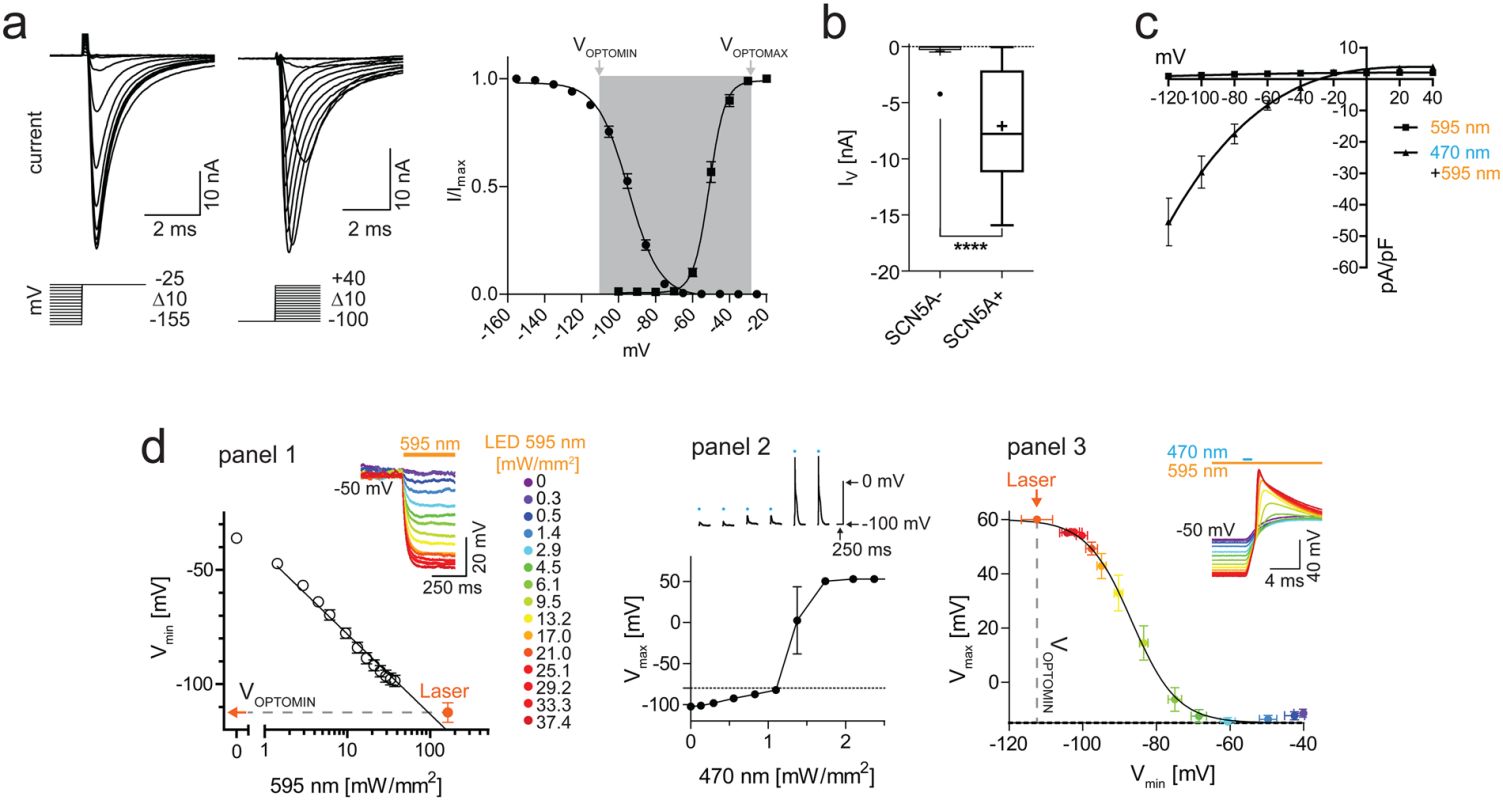
Defining the hNa_v_1.5-LiEp assay range. (**a**) Voltage-clamp. hNa_v_1.5 steady-state inactivation (left current traces and ● in plot, N = 35) and activation (right current traces and ◾ in plot, N = 16) characteristics (curves: Boltzmann sigmoidal with least squares fit). The optogenetically addressable voltage range is indicated by the grey underlay in the plot and limited by V_OPTOMIN_ (maximal ArchT-mediated hyperpolarization) and V_OPTOMAX_ (equivalent to V_rev_ ChR2), respectively. (**b**) Maximal voltage-gated currents in ChR2-βArchT expressing NG108-15 cells co-expressing hNa_v_1.5 channels (SCN5A + N = 56) and without (SCN5A−N = 15). (**c**) ChR2 peak and ArchT stationary photocurrent-voltage relationships triggered by 473/595 nm and 595 nm illumination, respectively (N = 7). (**d**) Current clamp. Panel 1: Light-tuning of the ArchT-mediated peak hyperpolarization (V_min_; logarithmic curve fit). Saturation at −112 mV (*P* = 163 mW/mm^2^, orange data point (N = 10), defining V_OPTOMIN_. Example traces shown with color coded 595 nm intensity (N = 13). Panel 2: Blue light intensity tuning of the ChR2-mediated hNa_v_1.5 peak potential (V_max_, N = 3), example voltage traces above. Panel 3: Dependence of the blue-light induced hNa_v_1.5 peak potential (V_max_) on the yellow light triggered hyperpolarization (V_min_). Error bars in x-axis according to panel 1 with identical color coded 595 nm intensity. hNa_v_1.5 steady-state de-inactivation was achieved with the laser subsequently used for LiEp experiments. curve: Boltzmann sigmoidal with least squares fit, N = 9.

The hNa_v_1.5 channel is the most challenging cardiac channel in terms of optical screening due to its fast kinetics and marked state-dependency. A drug-binding assay must cope with the channel’s millisecond activation and inactivation kinetics over a large voltage range (Fig. 1a, Suppl. Fig. S1a). For rapid bi-directional transmembrane voltage control, we constructed an improved optogenetic tandem protein^13^ combining ChR2 and ArchT (ChR2-EYFP-βArchT) to enable calibrated blue light-induced cell depolarization and yellow light-induced cell hyperpolarization (Suppl. Fig. S1b). We chose undifferentiated NG108-15 cells as recipient cells since they have been successfully used in previous experiments for high expression of tandem proteins^13^ and since they are devoid of endogenous fast voltage-gated currents (Fig. 1b; −0.40 ± 1.07 nA, N = 15). NG108-15 cells transfected with *pSCN5A* encoding the alpha subunit of the hNa_v_1.5 channel clearly developed voltage-step induced sodium currents (Fig. 1b; −7.09 ± 4.75 nA, N = 56, p < 0.0001). To determine the voltage-range of hNa_v_1.5 activity in physiological solution, that is the voltage range that needs to be modulated by optogenetic membrane potential control, we plotted the voltage dependence of hNa_v_1.5 activation and inactivation using VC (Fig. 1a). At potentials ≤ −100 mV over 50% of hNa_v_1.5 channels were de-inactivated and available (V_50_ = −94.7 mV) and at potentials ≤ −135 mV all hNa_v_1.5 channels were available. Membrane depolarizations above −65 mV activated a hNa_v_1.5 current that maximized at depolarizations ≥ −25 mV (V_50_ = −51.0 mV). We next co-transfected *pcDNA3.1-ChR2-EYFP-βArchT* and *pSCN5A*. Light induced ChR2 (−17.5 ± 8.3 pA/pF at −80 mV) and ArchT current densities (1.8 ± 1.0 pA/pF at −40 mV) confirmed functional expression of both, ChR2 and ArchT, which was previously shown to be stoichiometric^13^ (Fig. 1c, Suppl. Fig. S1b). The resting membrane potential of transfected NG108-15 cells was −42.3 ± 9.7 mV (N = 43), a voltage at which all hNa_v_1.5 channels are inactivated (Fig. 1a). By stepwise increasing the yellow light intensity, NG108 cells were increasingly hyperpolarized, reaching −98 ± 8.8 mV (N = 13) by maximal LED (595 nm, 37.4 mW/mm^2^) and −112.4 ± 13 mV (N = 10) by laser (593.5 nm, 163 mW/mm^2^) illumination (Fig. 1d panel 1). The plateau of hyperpolarization at −112 mV defines the lower limit of the LiEp assay (V_OPTOMIN_) and is a result of the ArchT activity approaching zero at −120 mV (Fig. 1c). The maximum hNa_v_1.5 action potential (AP) amplitude of +55 ± 2.0 mV (N = 9) was elicited by applying 2 ms blue light (473 nm) pulses with an intensity of ≥1.5 mW/mm^2^ on maximal 163 mW/mm^2^ 595 nm background illumination (Fig. 1d, panel 2). A de-inactivation curve of hNa_v_1.5 currents was again plotted, this time from the blue-light induced hNa_v_1.5 AP amplitudes recorded in current-clamp (CC) upon increasing levels of yellow background illumination. This “optical” curve (V_50_ = −86.7 ± 1.0 mV, R^2^ = 0.91, Boltzman fit; Fig. 1d panel 3) aligned well with the de-inactivation curve generated from VC data (Fig. 1a), despite the known nonlinear relation of channel current and AP amplitude^22^ (see Suppl. Fig. S1c and Suppl. Note 1). Consequently, hyperpolarization to −112 mV (corresponding to 163 mW/mm^2^ 595 nm illumination) sufficed to de-inactivate sufficient channels for a maximal blue-light induced hNa_v_1.5 AP. These data demonstrate that hNa_v_1.5 channel states can be modified by tuning the yellow light intensity. The reversal potential of the ChR2 peak current under 595/473 nm co-illumination was −28.5 mV (Fig. 1c), defining V_OPTOMAX_ of the LiEp interrogation window (Fig. 1a) and lying well above the hNa_v_1.5 activation threshold.

In summary, the light-tunable de- and hyperpolarization window of ChR2-EYFP-βArchT-expressing NG108-15 cells (~−112 mV to −28 mV) was sufficiently broad to effectively interrogate all hNa_v_1.5 conformational states.

### Optical and pharmacological hNa_v_1.5 interrogation

**Figure 2:**
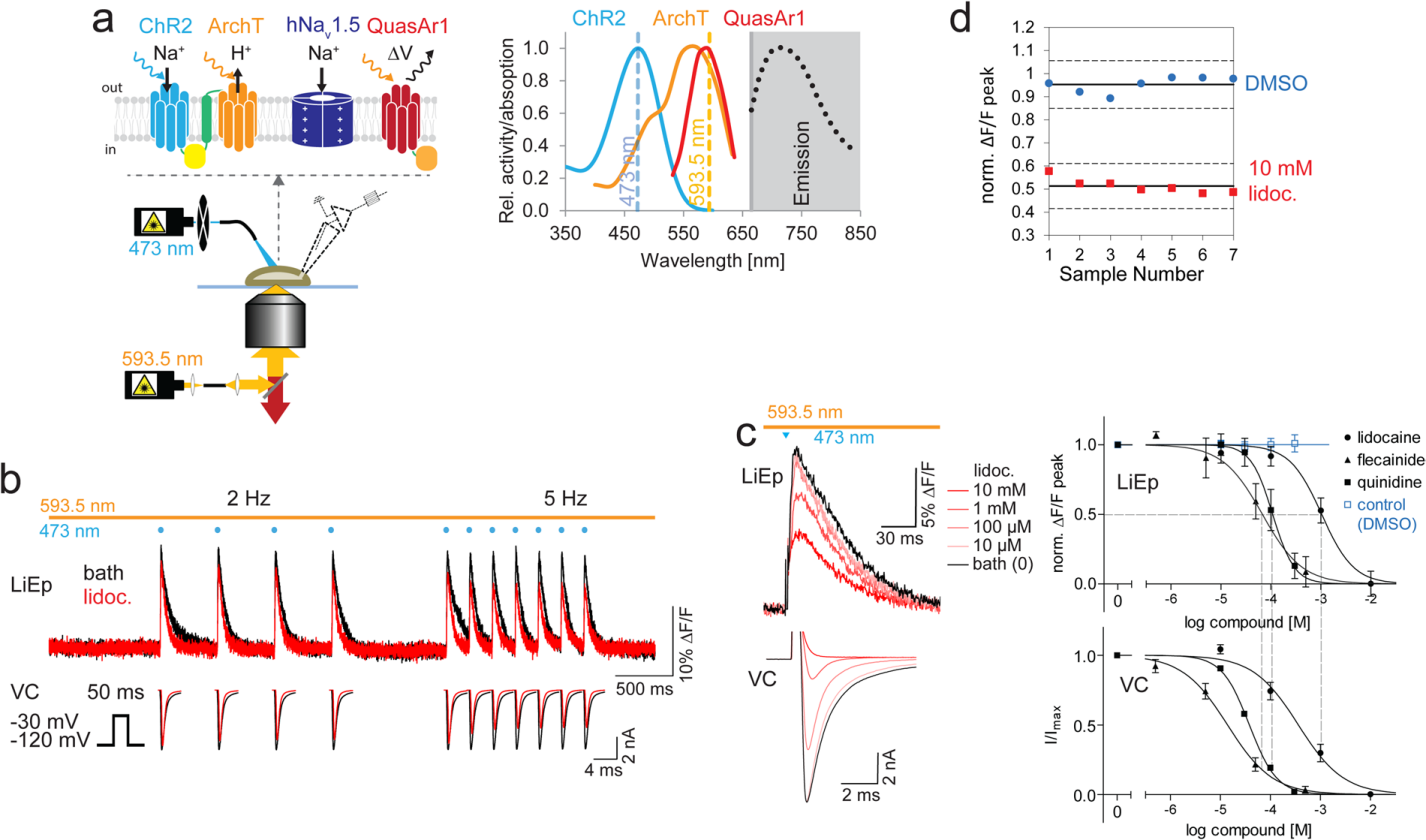
LiEp on hNa_v_1.5. (**a**) Schematic depiction of the cellular expression system (transient co-expression of QuasAr1-mOrange2-P2A-ChR2-βArchT and hNa_v_1.5), the imaging set-up and the relevant optical spectra^14,15,23^. (**b**) Frequency-dependent (2 Hz and 5 Hz pacing) block of hNa_v_1.5 by lidocaine (300 μM) recorded optically by LiEp (top) or conventionally by voltage-clamp (VC, bottom). (**c**) Example traces (left) for dose-dependent pharmacological action of lidocaine on hNa_v_1.5 channels evaluated by LiEp (top) and voltage-clamp (VC, bottom, step protocol as in (**b**)). Hill curves (right) were derived from ∆F/F peak values (top) and current amplitude (bottom), respectively. Half-maximal inhibition (IC_50_) values obtained by LiEp are indicated by dashed lines for comparison. A DMSO control trial is shown in blue with identical DMSO concentrations as used in the quinidine drug trial (0.01‰, 0.1‰, 1‰, 1%; N = 11). (**d**) LiEp signal window analysis within a single culture batch. ∆F/F peak readouts normalized to bath solution under negative control (DMSO) and positive control (10 mM lidocaine) conditions for 7 samples each (Z’ = 0.54, mean ± 3 × s.d., solid, resp. dashed lines).

To design an all-optogenetic LiEp assay, we implemented the archeorhodopsin-3 derived red-shifted genetic voltage indicator QuasAr1, shown to possess millisecond kinetics and small spectral overlap with ChR2^23^. We linked QuasAr1 N-terminally to the optogenetic tandem construct (QuasAr1-2A-ChR2-EYFP-βArchT) in order to achieve virtually “fixed” expression ratios of the three optogenetic tools. Since the QuasAr1 excitation spectrum almost completely overlaps with that of ArchT (Fig. 2a) we co-activated ArchT and QuasAr1 with the yellow laser and superimposed blue light pulses for ChR2 activation, triggering hNa_v_1.5 activity (Fig. 2b). This superimposed lighting protocol, although devoid of separable control of imaging and hyperpolarization, has the advantage that the ChR2 ground state is continuously re-populated, maximizing the ChR2 peak photocurrent under repeated illumination^24^ (Suppl. Fig. S1b).

We first tested LiEp-based pharmacological screening of hNa_v_1.5 with the use-dependent sodium channel blocker lidocaine^25^. We imaged the fluorescence changes of selected ChR2-ArchT-QuasAr1-expressing NG108 cells and concurrently patched one of the cells for CC recordings (Suppl. Fig. S2a). At 2 Hz blue light activation, low-dose (300 μM) lidocaine cumulatively reduced the ΔF/F amplitude with increased block at higher (5 Hz) pacing frequencies, a hallmark of use-dependency (Fig. 2b). A steady-state block developed after the 3^rd^ to 4^th^ light pulse, equally in LiEp and VC recordings. We were unable to test the lidocaine use-dependency more rigorously since our holding potential was fixed to −112 mV with the required high QuasAr1 imaging light intensity (163 mW/mm^2^)^23^ preventing fine-tuned population of different hNa_v_1.5 states.

In the next set of experiments, we tested the quality of dose-response data delivered by LiEp compared to VC. According to standard hNa_v_1.5 VC protocols^8^ we used a sequence of seven blue (2 ms) light pulses delivered at 5 Hz and averaged the last three hNa_v_1.5 APs to ensure full hNa_v_1.5 block (Suppl. Fig. S2a). The QuasAr1 ∆F/F peak fluorescence decreased concomitantly with the hNa_v_1.5 current amplitude in VC under increasing lidocaine concentrations (Fig. 2c). The lidocaine block was fully reversible (washout 88.4 ± 6.3%, N = 14, p < 0.0001), was not apparent in cells that had not been co-transfected with *pSCN5A* (94.2 ± 6.0%, N = 6, p < 0.0001) and no block developed when the solvent DMSO was applied alone (95.0 ± 8.4%, N = 16 in 1% DMSO, p < 0.0001; Suppl. Fig. S2d). Consequently, LiEp generated a large signal window with a Z’ value of 0.542 (DMSO: 95.3 ± 3.4% *vs*. 10 mM lidocaine 51.4 ± 3.3%, N = 7 each, Fig. 2d). To fit a Hill curve to the dose-response data, we determined the effective hNa_v_1.5 activity by subtracting the underlying ChR2 ∆F/F value (53.1 ± 11.0%, N = 14) measured under saturating lidocaine concentrations (10 mM, Suppl. Figs S1c, S2d,e). The Hill curves for lidocaine action on hNa_v_1.5 acquired with LiEp and VC were highly similar (Fig. 2c, Table 1). To confirm the validity of the LiEp protocol, we ensured signal stability under control conditions (DMSO) and evaluated two additional antiarrhythmic drugs, quinidine and flecainide. Also here the dose-response curves generated by LiEp and VC agreed well (Fig. 2c, Table 1, Suppl. Note 1).

**Table 1:** Comparison of electrophysiological and LiEp pharmacological quantification.

VGC Voltage sensor	Drug	All-optical				Voltage clamp			Current clamp		
		IC_50_ [µM]	Hill slope /R^2^	N	Z′	IC_50_ [µM]	Hill slope	N	IC_50_ [µM]	Hill slope	N
hNa_v_1.5 QuasAr1	Lidocaine	1040	−1.36/0.76	9	0.54	363	−0.96	7	—		
	Quinidine	108	−2.00/0.58	10	—	38	−1.59	5	—		
	Flecainide	67.5	−1.09/0.62	9	—	13.9	−0.96	8	—		
hERG Di-4-ANBDQPQ	Quinidine	15.4	−1.02/0.92	5	0.54^†^	1.27	−0.91	6	6.65*	−1.79	2
	Astemizole	1.58	−1.48/0.93	5	0.61^†^	0.61^$^	−0.81	7	1.17*	−1.77	3
	Terfenadine	3.62	−1.19/0.74	6	—	0.63	−0.92	5	1.85*	−1.02	5
hERG BeRST1	Quinidine	4.56	−1.03/0.96	3	0.69^†^	—	—		—		
	Astemizole	0.67	−1.62/0.99	4	0.66^†^	—	—		—		
	Terfenadine	1.20	−1.10/0.90	4	0.69^†^	—	—		—		
hK _v_1.5 RH421	DPO-1	0.09	−1.87/1.00	5	0.55	0.06	−1.80	5	—		

^†^maximally applied drug concentration against 0.1% DMSO, *fit to normalized difference in negative peak membrane potential of hERG hyperpolarization and steady-state voltage as shown in Fig. 4a, ^**$**^values less sensitive than generally reported in literature^31,57^, potentially due to different frequency of depolarization and shorter depolarization pulses.

The tested drugs did not interact with the optogenetic proteins, apparent from a positive ∆F/F peak amplitude at full block (10 mM lidocaine: 0.09 ± 8.9% N = 9; 500 μM flecainide: 8.6 ± 10.8% N = 9; 300 μM quinidine: 12.9 ± 9.1% N = 10, mean ± s.e.m) and no change in baseline QuasAr1 fluorescence (normalized to F_baseline_ in bath solution: 1% DMSO control 1.07 ± 0.15 N = 11, lidocaine 1.09 ± 0.11 N = 9, quinidine 1.12 ± 0.07 N = 10, flecainide 1.11 ± 0.10 N = 9; p = 0.705, one-way ANOVA). However, saturating quinidine concentrations, as described previously^26^, significantly reduced the amplitude of the ChR2 photocurrents (Suppl. Fig. S3, Suppl. Table S1) but did not affect activation of hNa_v_1.5, since the ChR2 photocurrent was still large enough.

In summary, bi-directional dynamic LiEp screening robustly and reliably reports drug interactions on hNa_v_1.5 with comparable precision to parallel VC recordings.

### LiEp on hERG

Due to the primary importance of hERG safety profiling we also designed a LiEp-hERG assay. However, the high illumination intensity needed for QuasAr1 activation^23^ as well as the dim QuasAr1 fluorescence (Suppl. Fig. S3, Suppl. Table S2) are not compatible with present fluorescence plate readers. We therefore explored in the following the potential of the organic voltage-sensitive dyes Di-4-ANBDQPQ^27^ and BeRST1^28^, which operate under significantly less light and give better signal-to-noise fluorescence ratios (Suppl. Fig. S3, Suppl. Table S2). Another advantage of organic voltage indicators is their uniform targeting to the entire cell layer, enabling fluorescence readout across the whole field of view.

### Imaging with Di-4-ANBDQPQ

**Figure 3:**
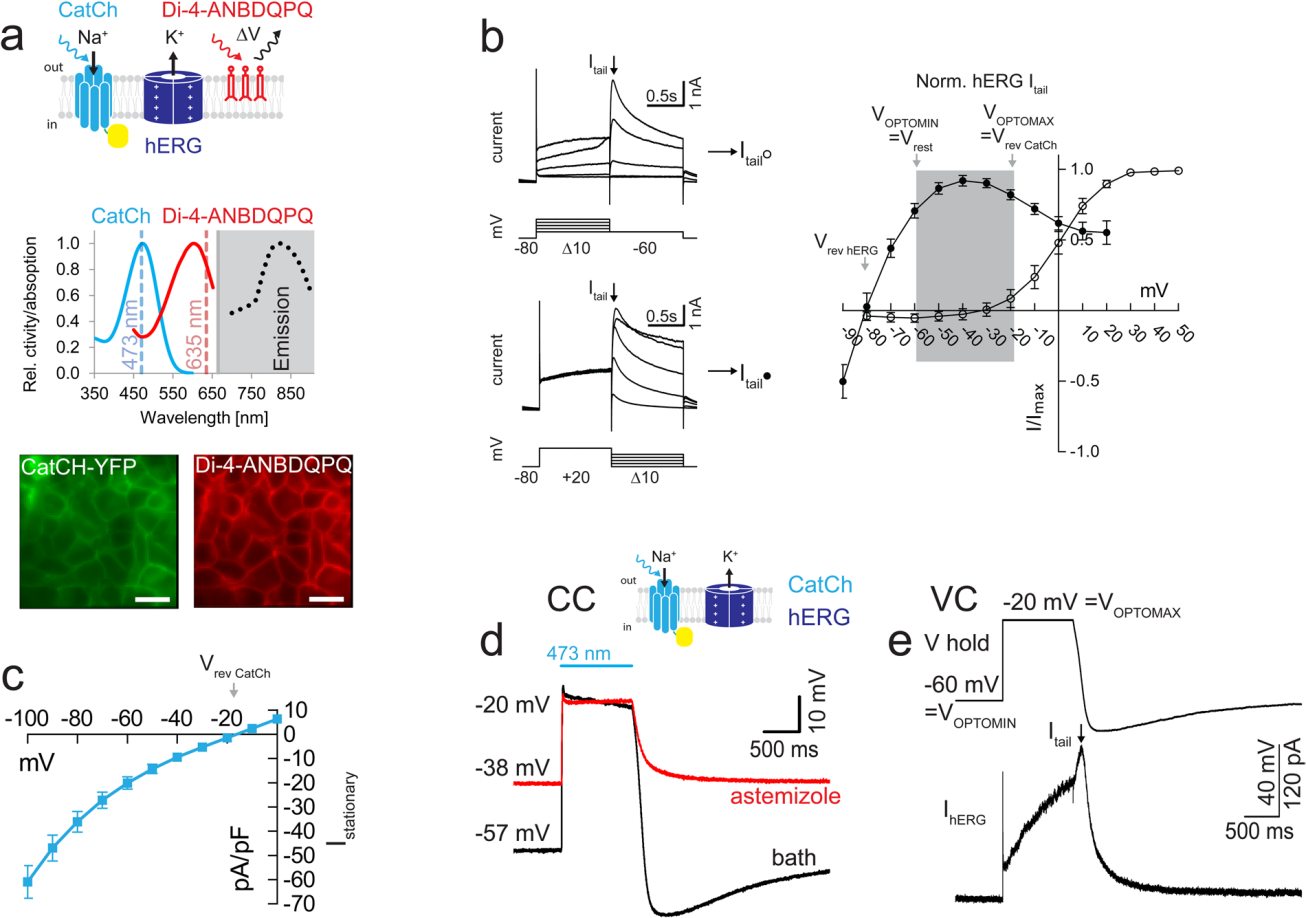
Defining the hERG-LiEp assay range. (**a**) Depiction of the cellular system (top), relevant spectra^14,27^ (middle) and photomicrograph of the fluorescent cell layer taken with the imaging CCD camera (bottom). Scale bars 20 µm. (**b**) Left: classical two-step voltage-clamp protocols for activation and inactivation of the hERG tail current (I_tail_) and corresponding plot (○ activation, N = 8; ● de-activation, N = 10). V_OPTOMIN_ was defined by the HEK293-CatCh cellular resting membrane potential, V_OPTOMAX_ by the CatCh reversal potential (compare panels c&d) and the grey underlay visualizes the LiEp-accessible voltage-control window. (**c**) CatCh stationary current-voltage relationship in HEK-CatCh cells (*P*_473_ = 1 mW/mm^2^, N = 15). (**d**) Current-clamp (CC). 473 nm “light-off” triggered hERG-hyperpolarization below the resting membrane potential (black trace) that is abolished by 30 μM astemizole (red trace). Note that astemizole also elevated the resting membrane potential. (**e**) Voltage-clamp (VC). The membrane potential waveform recorded in d (top) activates the hERG tail current (bottom).

We transiently introduced *pcDNA3.1-KCNH2* encoding the alpha subunit of the human hERG channel into a stable optogenetic HEK293-ChR2(L132C) “CatCh” cell line^29^ and measured the field of view compound response to a blue light pulse with the far-red voltage sensor Di-4-ANBDQPQ (λ_max_ 603 nm, Fig. 3a)^27^. We defined the voltage range of hERG activity by using standard voltage-step protocols^30,31^ (Fig. 3b). Activation of the hERG tail current required a depolarization step above −30 mV, saturating at steps above +30 mV (N = 10). hERG currents reversed at around −80 mV. hERG expressing HEK293-CatCh cells had a resting membrane potential of −58.2 ± 6.4 mV (N = 14), defining V_OPTOMIN,_ and CatCh stationary photocurrents reached −20.4 ± 8.6 mV (N = 8), defining V_OPTOMAX_ (Fig. 3b–d). To optically mimic the depolarization step applied in VC protocols, we employed a 1 sec blue laser pulse (1 mW/mm^2^, Fig. 3d) in CC. At the end of the light-triggered depolarization to −20 mV we observed a clear hyperpolarization before return to the resting membrane potential, which was abolished by the hERG blocker astemizole (30 μM). We subsequently used the light-triggered and resting membrane potentials as command voltages in a VC protocol to confirm the validity of the LiEp protocol (Fig. 3e). We delivered a 1 sec depolarization step to −20 mV (V_OPTOMAX_) followed by repolarization to −60 mV (V_OPTOMIN_) that was able to produce a (non-saturated) hERG outward tail current. Controlled channel closure could be improved by using the optogenetic tandem actuator ChR2-βArchT combined with a spectrally separable voltage indicator, which is not given for Di-4-ANBDQPQ.

**Figure 4:**
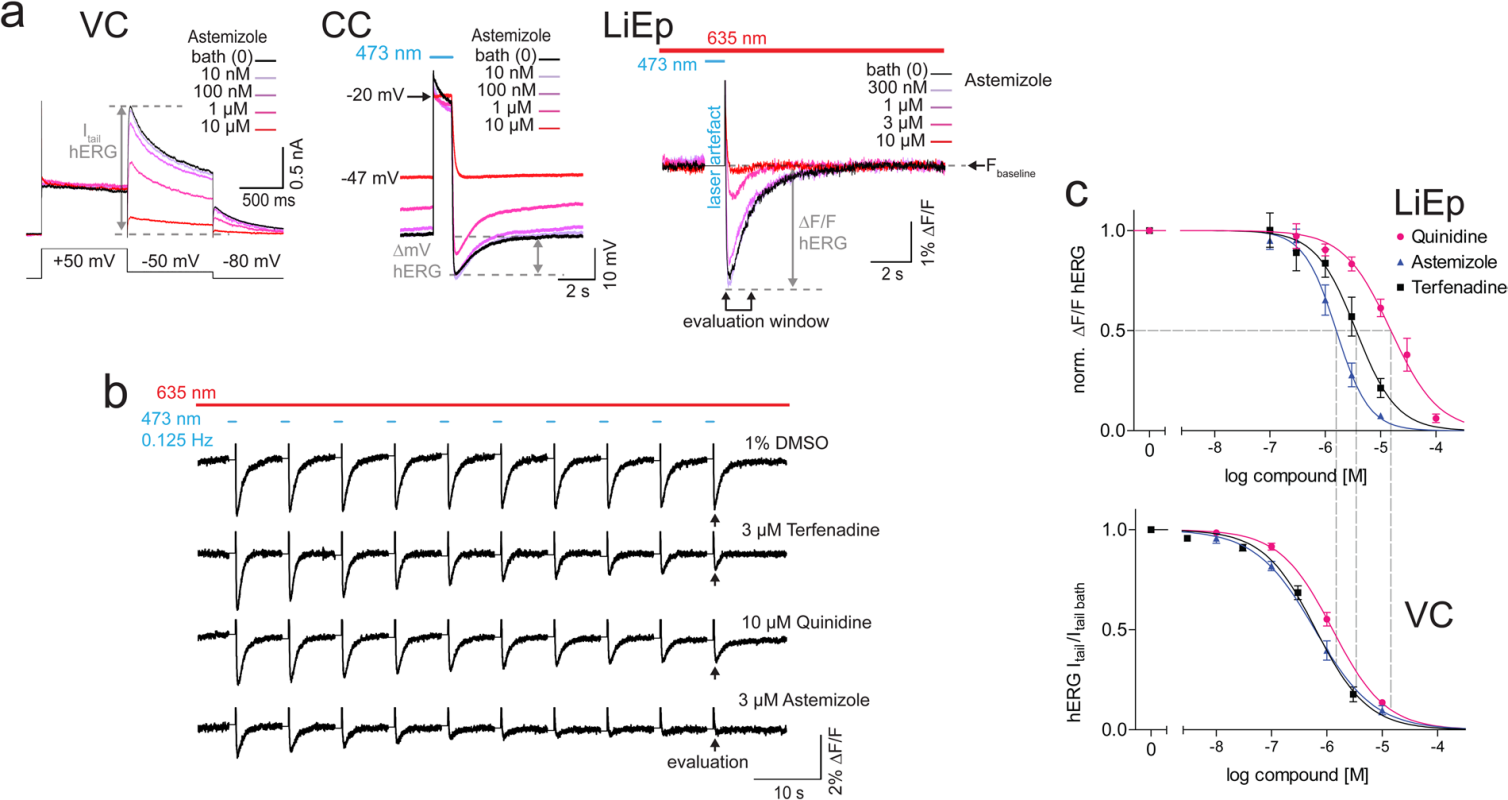
LiEp on hERG with Di-4-ANBDQPQ. (**a**) Astemizole-induced inhibition of hERG tail currents depicted for the three different measurement modes, voltage-clamp (VC), current-clamp (CC) and LiEp. The quantified signal amplitude for dose-response analyses is indicated for each method: I_tail_ hERG current amplitude in VC, ∆mV hERG hyperpolarization to resting potential in CC and ∆F/F hERG hyperpolarization to resting F_baseline_ for LiEp. The blue light cross-excitation of Di-4-ANBDQPQ masked the CatCh-depolarization in the LiEp fluorescence readout (laser artifact) and traces were therefore aligned to F_baseline_. (**b**) LiEp readout under control conditions (1% DMSO) or in the presence of hERG inhibitors. Cumulative block was assessed at the 10^th^ steady-state response (arrows). (**c**) Comparative dose-response relationships measured by LiEp and direct I_tail_ recordings in VC (bottom). Stippled lines indicate the IC_50_ values of the LiEp assay for better comparison.

Next, we compared pharmacological data of the antiarrhythmic quinidine and the antihistaminics astemizole and terfenadine derived from VC, CC and LiEp side-by-side (Fig. 4a–c). Blue light stimulation induced a substantial stimulus artifact in the Di-4-ANBDQPQ fluorescence signal and we therefore used the post-stimulus steady-state baseline fluorescence (after cessation of the hERG tail current) for trace alignment (Fig. 4a). We applied ten consecutive blue light pulses and used the last ∆F/F value when a steady-state cumulative block was reached (Fig. 4b). After each 10-pulse stimulation protocol the imaging location had to be changed due to Di-4-ANBDQPQ toxicity, obvious from granulation of somata and cell shrinkage. All drugs reliably blocked hERG in a concentration dependent manner and in good agreement with VC (Fig. 4c, Table 1). Drug application also depolarized the resting membrane potential, representative for a basal hERG conductance (10 μM astemizole −43.9 ± 5.2 mV, N = 5, p = 0.0003; Fig. 4a).

### Imaging with BeRST1

**Figure 5:**
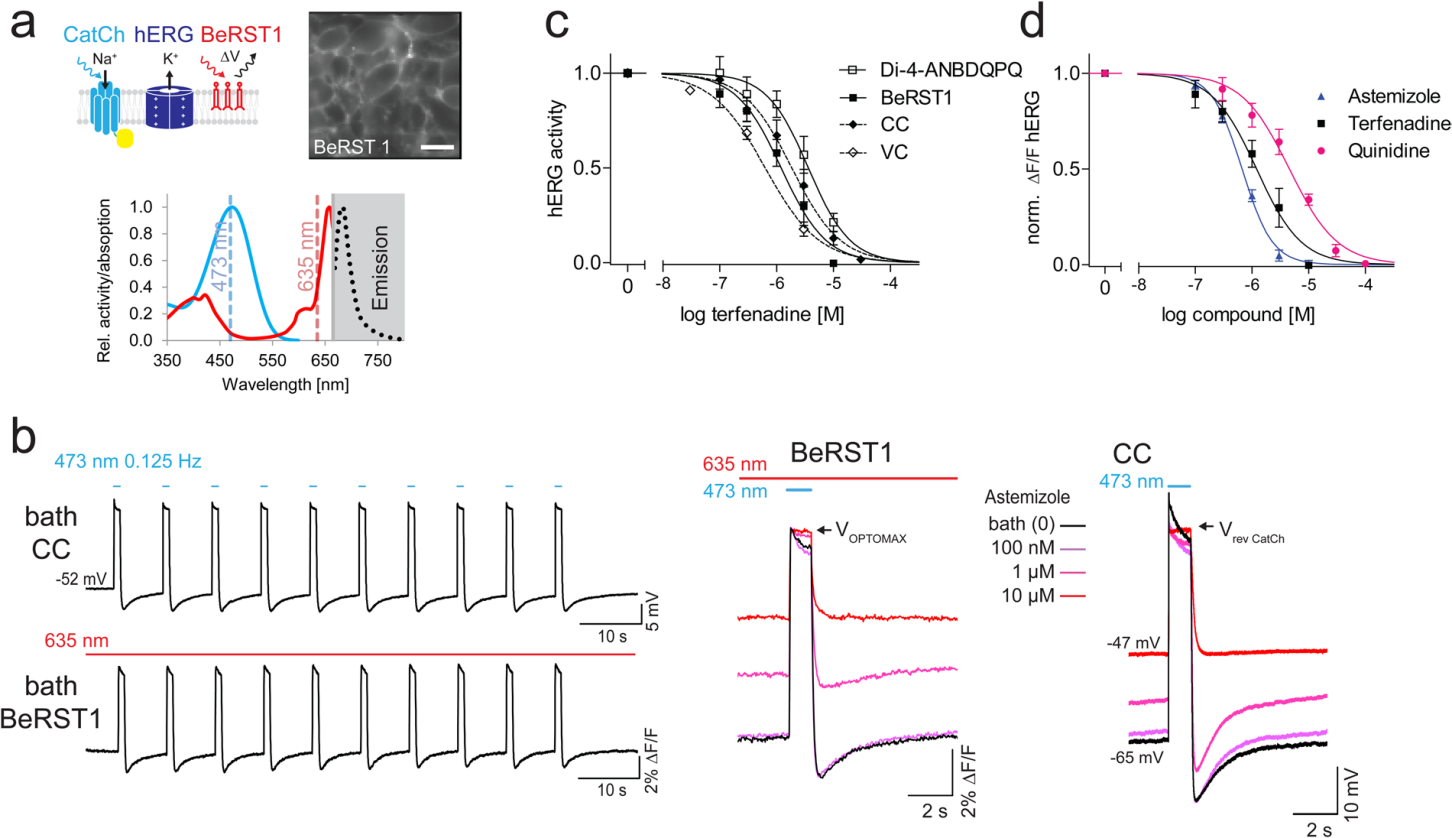
LiEp for hERG using BeRST1. (**a**) Cellular system, relevant spectra^14,28^ and BeRST1 labeled cells (scale bar 20 µm). Note that the 473 nm CatCh activation does not overlap with the BeRST1 excitation spectrum. (**b**) Comparison of current-clamp (CC, top) and LiEp (bottom) recordings in bath solution. Note that the blue light-induced CatCh depolarization is resolved in the LiEp (BeRST1) signal. Right: LiEp optical traces aligned to V_OPTOMAX_ and CC traces aligned to the CatCh reversal potential (V_rev_) under increasing concentrations of astemizole. Elevation of the resting potential with increasing astemizole concentrations is resolved in both modes. (**c**) Comparative quantification of hERG inhibition by LiEp/Di-4-ANBDQPQ, LiEp/BeRST1, current-clamp (CC) and voltage-clamp (VC) for the drug terfenadine. (**d**) Drug-response curves for astemizole, terfenadine and quinidine as obtained with LiEp/BeRST1.

Huang and colleagues (2016) recently designed a novel voltage-sensitive sulfonated-silicon-rhodamine-fluorophore-based dye, the Berkeley Red Sensor of Transmembrane potential (BeRST1) with a relatively narrow excitation spectrum and only 0.1% excitation at 473 nm^28^ (Fig. 5a). We tested if BeRST1 may enable cross-talk free LiEp and may overcome the phototoxicity issue of Di-4-ANBDQPQ. hERG block for all drugs gave an excellent signal window (Z’ ≥ 0. 66) and we detected no signs of phototoxicity allowing repeated data acquisition from the same imaging site. Compared to experiments with Di-4-ANBDQPQ, the CatCh mediated depolarization was resolved in the optical signal revealing the drug-induced membrane potential shifts analogously to CC (Fig. 5b). In addition, Hill curve fits further approached those from VC (terfenadine: Hill slope −1.10, IC_50_ 1.2 μM, N = 4; astemizole: 0.67 Hill slope −1.62, IC_50_ 0.67 μM, N = 4; quinidine: Hill slope −1.03, IC_50_ 4.56 μM, N = 3; Fig. 5c,d, Table 1).

Above data emphasizes the need for spectrally separable voltage sensors with better cell tolerability.

### LiEp on hK_v_1.5

**Figure 6:**
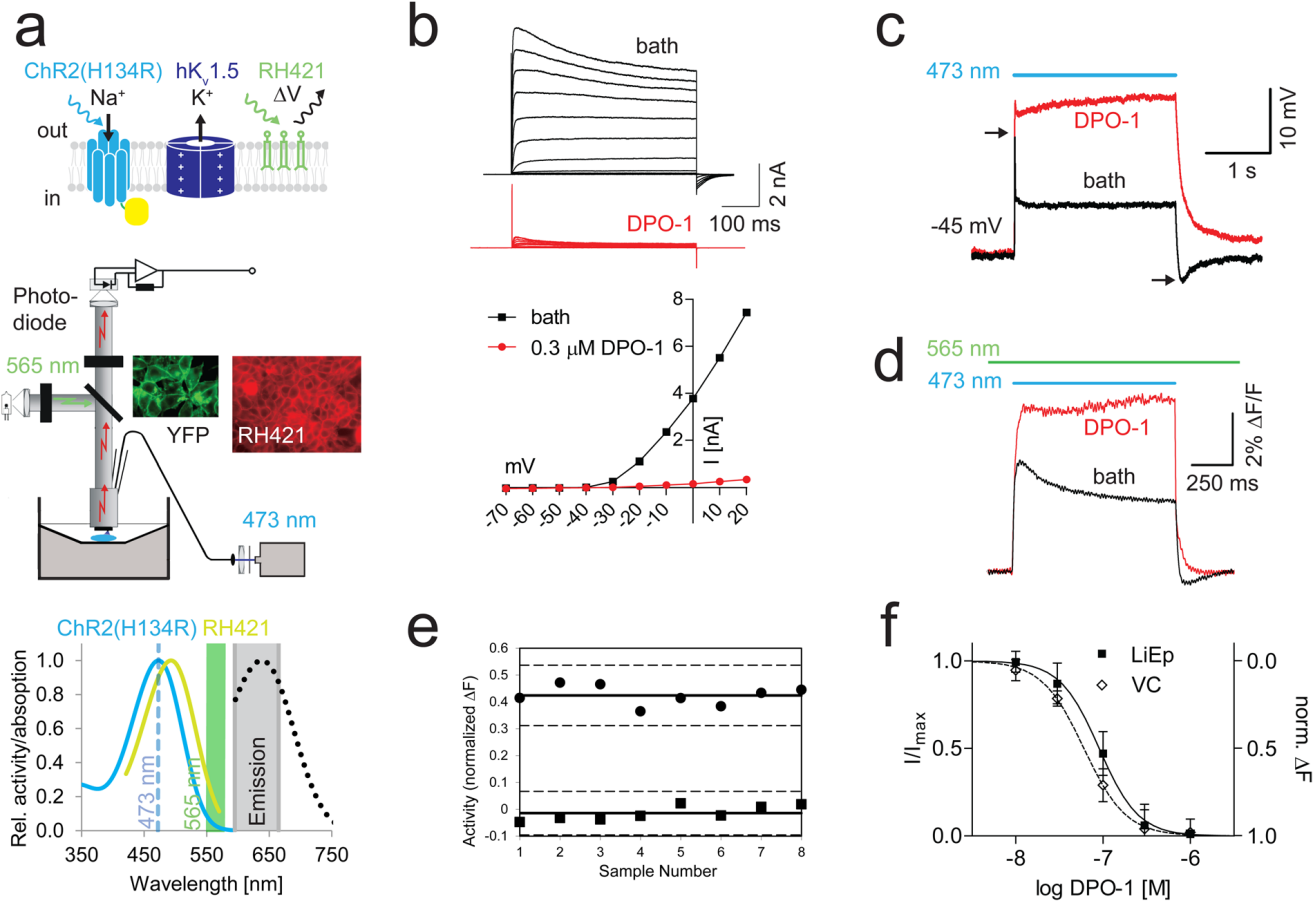
LiEp for hK_v_1.5. (**a**) Cellular system (top), imaging set-up (middle) and relevant spectra^14,56^. (**b**) 0.3 μM DPO-1 almost fully abolished hK_v_1.5 currents (top: voltage-clamp (VC), bottom: current-voltage relationship). (**c**) Example single-cell current clamp recording of the blue-light induced depolarization (black trace), which was increased upon block of hK_v_1.5 by 0.3 μM DPO-1 (red trace). Note the sharp hK_v_1.5-mediated transient at “light on” as well as the hK_v_1.5 induced hyperpolarization at “light off” (arrows). (**d**) Corresponding LiEp signal to (**c**) recorded from a HEK-*ChR2(H134R)-EYFP-IRES-hK*_*v*_*1.5* cell layer loaded with RH421 (as depicted in panel a). (**e**) Relative hK_v_1.5 activity changes measured from HEK-ChR2(H134R)-EYFP-IRES-hK_v_1.5 (●) cells and control HEK293-ChR2(H134R)-EYFP cells (◾) upon 0.1 μM DPO-1 application. Mean ± 3 × s.d. (solid, resp. dashed lines). (**f**) Comparison of single-cell VC and monolayer LiEp derived DPO-1 dose-response curves (N = 5), optical data was scaled to maximal and minimal effects.

We finally tested LiEp on the hK_v_1.5 potassium channel, an important anti-arrhythmic drug target. In light of assay standardization we generated a monoclonal doxycycline inducible TetON-HEK293-ChR2(H134R)-EYFP-IRES-hK_v_1.5 cell line, co-expressing the optogenetic actuator ChR2(H134R) with large stationary photocurrents^32^ and the voltage-gated potassium channel hK_v_1.5 (Fig. 6a). The selected clone had a resting membrane potential of −45 ± 5.6 mV (N = 13; V_OPTOMIN_). Additional optogenetic hyperpolarization was not required since hK_v_1.5 does not exhibit a depolarization block. VC recordings on stable TetON-HEK293-ChR2(H134R)-hK_v_1.5 clones revealed a clear potassium current with an activation threshold at −40 mV that was reversibly blocked (91 ± 8%, N = 5, Fig. 6b) by application of the open channel blocker and potential antiarrhythmic drug diphenylphosphine oxide (DPO-1, 0.3 μM)^33^. Under full hK_v_1.5 block the ChR2(H134R) steady-state reversal potential was at −7 mV (V_OPTOMAX_). We first performed blue light hK_v_1.5 actuation in CC to evaluate the readout signal. The ChR2(H134R) depolarizing current induced a secondary hyperpolarizing hK_v_1.5 current, which decreased the steady-state ChR2(H134R) depolarization by 20.56 ± 4.56 mV (N = 13, Fig. 6c). Besides the reduced steady-state amplitude of the light-induced voltage step, hK_v_1.5 channel activity was evident from the quick recovery of the light-induced ChR2 depolarization resulting in a sharp transient at “light on” as well as the hK_v_1.5 induced hyperpolarization at “light off” (Fig. 6c). To further demonstrate the tractability of LiEp, we explored yet another fast organic voltage sensor, RH421^34^ (Fig. 6a). Due to the significant spectral overlap of the activation spectra of RH421 and ChR2(H134R) we activated RH421 with narrow-band (565/24) green light at the red absorption flank that was too low in intensity (<0.01 mW mm^−2^) to significantly activate ChR2(H134R) (Fig. 6a). Since DPO-1 is an open channel blocker, we applied 1 second long 473 nm light pulses in imaging experiments and only analyzed the final 100 ms of RH421 fluorescence acquisition when a full block had developed (Suppl. Fig. S4). The recorded RH421 signal from the TetON-HEK293-ChR2(H134R)-hK_v_1.5 cell layer in response to DPO-1 application changed analogously to CC (Fig. 6d). When comparing the normalized fluorescence changes upon blue light stimulation in bath solution containing 0.3 μM DPO-1 between TetON-HEK293-ChR2(H134R)-hK_v_1.5 cells (∆F = 42.4 ± 3.7%, N = 8) and HEK-ChR2-YFP cells^35^ (∆F = 1.4 ± 2.7%, N = 8), LiEp delivered an excellent signal window (Z′ = 0.55) (Fig. 6e). Comparing data against recordings from untransfected HEK-ChR2-YFP cells allowed us to exclude unspecific DPO-1 effects on ChR2. We next applied LiEp to pharmacological characterization of DPO-1 and compared the concentration-response curves generated by VC and LiEp side-by-side using a 0.5 Hz stimulation protocol. The two concentration-response curves were highly similar (Fig. 6f; LiEp: IC_50_ 90 nM, Hill slope −1.94, N = 5; VC: IC_50_ 61 nM, Hill slope −1.81, N = 5) and agreed well with literature values^36^.

In summary, above experiments corroborate the suitability of LiEp in a plate reader format using stable cell lines.

## Discussion

LiEp is the first all-optical method meeting the temporal precision and dynamic transmembrane potential control required for high-content pharmacological drug characterization on VGCs and distinguishes itself from previously proposed optical methodologies^23,37–41^ by bi-directional optogenetic membrane potential control^13^. We demonstrated the tractability and reproducibility of LiEp by exchanging optogenetic actuators and voltage sensors to optically investigate the activity of various VGCs.

Our study focused on demonstrating the potential impact of optical drug screening rather than on new electrophysiological insights or novel drug evaluation. In these terms, LiEp produced excellent screening signal windows with Z′ values above 0.5, enabled optical detection of use-dependency, which had previously been demonstrated by other groups using optical protocols^11,38,40^ and closely reproduced IC_50_ and Hill coefficient values from parallel VC recordings, *i.e*. within 1 log unit or 1–4 fold difference, respectively, ranges well accepted in automated electrophysiology assays^42^. The small, yet systematic IC_50_ discrepancies between VC and LiEp measurements on hNa_v_1.5 channels arise from the known non-linear relation of channel current and membrane potential amplitude (see also Suppl. Note 1). The accuracy of the LiEp assay is remarkable given that, in contrast to the virtually unlimited signal amplitude of ionic channel currents in VC, a closed-loop circuit as is needed for a “true clamp” is not in place in LiEp. Optogenetic tools possess complex photocurrent kinetics and are unable to counteract the fast gating and high conductivity of VGCs. In addition the membrane capacitance also influences the voltage changes. The unclamped membrane potential, and hence the optical readout, is consequently restrained by the optogenetic reversal and ionic equilibrium potentials as well as the imaging light intensities. One interesting prospect of our study is that LiEp may ease measurements of fast Na_v_ channels at physiological temperature, which is highly challenging in VC since compensatory feedback is prone to “self-clamping” of the channel-expressing cell.

One drawback of optical compared to electric interrogation is, however, the difficulty to relate light-step protocols to well-defined potential-step protocols, which are ultimately needed for accurate VGC probing. A stable optogenetic cell line could, in principle, allow the experimenter to directly assign light intensity at a given wavelength to transmembrane voltage, by relating the reversal or resting membrane potentials to particular fluorescence values on the voltage sensor’s (linear) calibration curves (see Fig. 5b, Suppl. Fig. S5).

Optimally LiEp will be performed by 3D orthogonal optical stimulation, using a blue-light activated channelrhodopsin, a yellow light-activated ArchT and far-red activated voltage sensor with fully separable activation spectra. In our initial LiEp assays the spectral crosstalk of QuasAr1 with ArchT reduced the sensitivity of hNa_v_1.5 to lidocaine compared to literature values^8^ since the cells were depolarized to the resting membrane potential in the inter-imaging intervals when the yellow laser was turned off. This modified the lidocaine binding kinetics^43^. A genetic voltage indicator such as QuasAr1 has the ability to be stably integrated into a cell line, which would simplify the screening assay and reduce costs. Other non-pumping near infrared fluorescing Arch-variants have been created by directed evolution but have not yet been evaluated as voltage sensors^44,45^. One problem with QuasArs is their extreme light requirements that lie 3–4 log units above the maximum light intensities in currently available plate readers, making customized platforms necessary. In contrast, the ChR2(D156A) variant with similar light-sensitivity to the here used CatCh has already been successfully actuated in a FLIPR tetra® reader (Molecular devices) for optogenetic Ca_v_ channel drug screening with Ca^2+^ sensor (Fluo-8) readout^39^. The very slow off-kinetics of ChR2(D156A)^24^, however, are not suited for screening of rapidly-gated VGCs such as hNa_v_1.5, but CatCh could be used instead. And voltage sensors are generally less sensitive than Ca^2+^ indicators. The development of spectrally separable and light-sensitive voltage sensors with fast temporal resolution will facilitate the adaptation of LiEp in a plate reader format.

For the LiEp platform to be reliable, compounds should not significantly interfere with the functionality of the optogenetic actuators or sensors. As reported previously by others^38,40^ we found no dramatic drug effects on the photocurrent amplitudes or kinetics of the employed optogenetic tools nor did the drugs affect the baseline fluorescence of the voltage sensors (Suppl. Fig. S3, Suppl. Table 1). One exception was quinidine, which, if applied at saturating doses, significantly reduced the ChR2 photocurrents in line with previous reports of photocurrent inhibition in *C. reinhardii* that endogenously expresses ChR2 and other opsins^26^. The quality of the LiEp assay remained, however, unaffected. A drug-channel interaction is easily detected in the LiEp assay when the ΔF/F signal falls below the ChR2 ΔF/F value (see Suppl. Fig. S2).

Present optical VGC screening techniques typically manipulate the membrane potential artificially, by increasing extracellular potassium to depolarize the cells^46^ and introducing a rectified potassium leak current, usually through Kir2.1, to hyperpolarize the cells^37,38,47^. Kir 2.1 modulates cell growth^48^ and is a CiPA safety target potentially susceptible to drug interactions^49^. Solution exchange decreases the assay’s temporal resolution and a changing extracellular ion composition can affect channel gating through selectivity filter interactions^50^. For all above reasons optogenetic membrane potential toggling through ArchT proton pumping and ChR2 cation conductance is a major advantage and enables contact-free, millisecond VGC interrogation under physiological conditions. The main advantages of LiEp over commercially applied fluorescence assays such as FLIPR^12,51,52^ and VIPR^10,46^ or the combined optical-electric E-VIPR^11^ are (i) the millisecond activation, deactivation and readout kinetics on the timescale of VC enabling the induction and study of transient and rapidly inactivating channels, (ii) the ability to run calibrated light-step protocols to differentially interrogate VGC conformational states and (iii) the possibility for repetitive stimulation to study use-dependency. Although plate readers are yet unable to deliver millisecond-switchable multi-wavelength excitation and possess readout latencies typically incompatible with fast-acting VGCs, the scalability of all-optical optogenetic assays has been shown using microscope-based platforms carrying high aperture optics combined with movable stages holding multi-well plates^38,40^.

In summary, we have shown that LiEp is a simple, fast and versatile technique fulfilling crucial criteria for adaptation into a high-throughput screening platform^53^. We showed that LiEp is compatible with a palette of VGCs, optogenetic actuators, voltage sensors and cell lines and in combination with suitable optical hardware may pose an alternative to complicated and costly electrophysiological platforms for future high-throughput screening. In particular, LiEp may enable large scale early phase VGC isoform specific profiling and can complement the recently proposed optical iPSC cardiomyocyte technologies in the framework of the CiPA initiative^40,41^.

## Methods

### Plasmids and molecular biology

Expression was in all constructs driven by the cytomegalovirus (CMV) promoter. *pcDNA3.1-SCN5A (pSCN5A)* encoding the alpha subunit of the human hNa_v_1.5 channel was a kind gift from Bela Kelety (IonGate GmbH). *pcDNA3.1- KCNH2* encoding the alpha subunit of the human hERG (K_v_11.1) channel was a kind gift from Hugues Abriel (University of Bern). The light-driven chloride pump NpHR from pcDNA3.1-ChR2-EYFP-βNphR^13^ was replaced by the light-driven proton pump ArchT^15^ (kindly supplied by Ed Boyden, MIT) to construct the vector *pcDNA3.1-ChR2-EYFP-βArchT*. QuasAr1-mOrange-P2A was amplified from FCK-Optopatch1^23^ (Addgene: 51695; F: 5′-CTATGGATCCACCA-TGGTAAGTATCGCT-3′, R: 5′-CTATGGATCCGGGACCGGGGTTTTCTTCCA-3′) and inserted in front of ChR2 in *pcDNA3.1-ChR2-EYFP-βArchT* using the BamHI restriction site, resulting in *pcDNA3.1-QuasAr1-mOrange2-P2A-ChR2-EYFP-βArchT*. EYFP served in all constructs for the visualization of successful expression in the receptive cell lines. VGC plasmids without EYFP were co-transfected with optogenetic EYFP-labeled constructs to identifiy transfected cells.

### Cell lines and culture conditions

Using effectene transfection (QIAGEN, Hilden, Germany) a stable inducible HEK293 cell line (TetON-HEK293-ChR2(H134R)-EYFP-IRES-hK_v_1.5) was created that doxycycline dependently co-expressed *ChR2(H134R)-EYFP* and *hK*_*v*_*1.5* from an internal ribosome entry site (IRES) using Clontech’s Tet-On 3 G system (Clontech, Saint-Germain-en-Laye, France). Undifferentiated NG108-15 neuroblastoma (ATCC® HB-12317^TM^) and HEK293 (ATCC® CRL-1573^TM^) cells were cultured in Dulbecco’s Modified Eagle Medium (Sigma Aldrich) supplemented with 10% fetal calf serum (FCS), 10 U/ml penicillin, 100 µg/ml streptomycin and 1 mM L-Glutamin (Millipore) at 37 °C with 5% CO_2_. HEK-CatCh cells^29^ were maintained under neomycin (750 μg/ml, Promega) selection, the TetON-HEK293-ChR2-hK_v_1.5 cell line under combined neomycin (800 μg/ml, Promega) and hygromycin (600 μg/ml, Sigma) selection.

For hNa_v_1.5 experiments, 1.6 × 10^5^ NG108-15 cells were plated on poly-L-ornithine (Sigma Aldrich) coated 22 mm glass coverslips in 12 well plates. *pcDNA3.1-ChR2-EYFP-βArchT* or *pcDNA3.1-QuasAr1-mOrange2-P2A-ChR2-EYFP-βArchT* were co-transfected with *pcDNA3.1-SCN5A* at a 2:3 molar ratio, using the Lipofectamin 2000 transfection kit (Life Technologies). For hERG experiments, 4 × 10^6^ HEK293-Catch cells^29^ were plated in a 10 cm diameter culture plate and transfected with *pcDNA3.1-KCNH2* using calcium phosphate precipitation^54^. 24 hrs after transfection, cells were replated in 12 well plates at densities of 4 × 10^5^. For hK_v_1.5 experiments, 5 × 10^4^ TetON-HEK293-ChR2(H134R)-EYFP-IRES-hK_v_1.5 cells were seeded in 24-well plates on poly-L-lysine coated coverslips.

Expression in TetON-HEK293-ChR2(H134R)-hK_v_1.5 cells was induced by adding 1 μg/ml doxycycline (Pfizer) to the culture medium 48 hrs before experiments. The culture medium was in all cases supplemented with 1 µM all-trans retinal (Sigma Aldrich) 12 hours prior to an experiment. Experiments were performed 24–48 h post transfection. All cell lines and LiEp assay settings are summarized in Table 2.

**Table 2:** LiEp assay settings.

Cell lines	VGC	Imaging sensor	Optogenetic actuator	Voltage sensor	Light (λ (nm)/*P* (mW/mm^2^)	Δt_473_ (ms)
NG108-15	hNa_v_1.5*	CCD camera	ChR2^14^* ArchT^58^*	QuasAr1^23^*	473/2 593.5/163	2
HEK293-CatCh-EYFP	hERG*	CCD camera	ChR2(L132C)^29^ CatCh	Di-4-ANBDQPQ^27^	473/1 635/5	1000
HEK293-CatCh-EYFP	hERG*	CCD camera	ChR2(L132C)^29^ CatCh	BeRST1^28^	473/0.8 635/20	1000
TetON-HEK293-ChR2 (H134R)-EYFP-IRES-hK_v_1.5	hK_v_1.5	Photodiode	ChR2(H134R)^32^	RH421^34^	473/0.7 656/24/<0.01	1000

***P*** given at sample, Δ**t**_**473**_ duration of blue light pulse. *transient expression.

### Whole cell patch clamp recordings

All electrophysiological recordings were performed at room temperature on an inverted Zeiss Axiovert 35 M microscope using borosilicate glass pipettes (Harvard Apparatus GC150F-10) pulled with a Zeitz DMZ-Universal puller with resistances ranging from 3 to 6 MΩ. The pipette solution contained (in mM): 123 K-Gluconate, 7 KCl, 1 MgCl_2_, 5 Na_2_-ATP, 10 EGTA, 10 HEPES; pH 7.35 (KOH), 285–290 mOsm. The bath solutions contained (in mM) for hNa_v_1.5 and hK_v_1.5 experiments: 135 NaCl, 3 KCl, 1 MgCl_2_, 2 CaCl_2_, 10 D-Glucose, 5 HEPES (pH 7.3) and for hERG experiments according to Chen *et al*.^30^: NaCl 130, CH_3_COONa 2.8; KCl 4, MgCl_2_ 1, CaCl_2_ 1, D-Glucose 10, HEPES 10 (pH 7.4). Liquid junction potentials were corrected for all experiments and series resistance compensation was applied for hNa_v_1.5 characterization. Signals were amplified with an Axopatch 200B amplifier, low pass filtered at 5 kHz and digitized at 50 kHz with an Axon Digidata 1440 A. Data acquisition and analysis were performed using pClamp software (Molecular Devices, Biberach, Germay). Drugs were dissolved and stored at −20 °C as high concentrated stocks in ddH_2_O (lidocaine: Sigma-Aldrich), DMSO (astemizole: Tocris; quinidine: Sigmal Aldrich) or ethanole (terfenadine and DPO-1: Tocris; flecainide: Abcam). In all experiments drugs were applied by gravity solution flow at increasing concentrations followed by 4 min washout with bath solution.

Voltage-clamp step protocols are given in suppl. Fig. S6. Whole-cell current-clamp (CC) experiments were performed with 0 pA holding currents. For ChR2-EYFP-ArchT light tuning curves (Fig. 1d), a tunable pE-4000 LED system (CoolLED Ltd. Andover UK) and a 593.5 nm shutter controlled diode-pumped solid state laser (Pusch Opto Tech GmbH) were used. τ_ON_ and τ_OFF_ values were determined with a single exponential fit (Chebyshev method) in Clampfit:

1 $$f(t)={\rm{A}}\ast {e}^{-t/\tau }+C$$

ChR2 current-voltage relationships were fitted with second and third order polynomial functions (extra sum-of-squares F test, simpler model selected if p > 0.05) and the best fit chosen for calculation of the reversal potential for f(x) = 0.

### LiEp activation, imaging processing and data analysis

All experiments were conducted at room temperature. For optogenetic activation we used either diode-pumped solid-state (DPSS), shutter-controlled (Uniblitz LS6ZM2, Vincent Associates) 473 nm and 593.5 nm lasers (Pusch Opto Tech GmbH) focused into optic fibers (Thorlabs), whereas the 473 nm laser was mainly used for channelrhodopsin activation and focused into a 400 μm diameter optic fiber the tip of which was directly placed into the bath proximal to the cells. Alternatively we used a tunable LED array (pE-4000, CoolLED Ltd. Andover UK) connected to the fluorescence port of the microscope. Details about the imaging settings such as light intensities, wavelengths and detectors are given in Table 2.

### hK_v_1.5 – RH421

HEK293-ChR2(H134R) cells on coverslips were incubated in 5 μM RH421 (*N*-(4-Sulfobutyl)–4-(4-(*p*-(dipentylamino)phenyl)butadienyl)-pyridinium inner salt; Life Technologies)^34^ for 10 min in the dark, followed by a brief wash in bath solution and transferred into a perfusion chamber mounted on a fluorescence upright Zeiss Axioskop 2FS microscope (Zeiss) equipped with a 40× water immersion objective (numerical aperture = 0.8; Fig. 6a). Fluorescence was excited by a 100-W tungsten lamp using a 565DF24 excitation filter, a 630DF69 emission filter, and a 585DRLP dichroic mirror (AF Analysentechnik, Tübingen, Germany) and whole-field fluorescence detected with a PIN-022A photodiode (United Detector Technologies, Brignoles, France) mounted to the microscope camera port (Fig. 6a). 473 nm light was passed through a heat absorption filter (KG filter, Schott), which removed long-wavelength stray light that increases background noise at the photodiode. Photodiode signals were amplified by a variable-gain low-noise current amplifier (Model DLPCA-200, FEMTO Messtechnik), digitized at 2 kHz with an Axon Digidata 1200 and acquired and analyzed using pClamp9 software (Axon Instruments).

Optical traces were started 5 s before and stopped 5 s after 473 nm laser pulses. The 473 nm stimulation artifacts were removed and the baseline corrected for RH421 bleaching by linear baseline subtraction in OriginLab software and the raw fluorescence trace inverted (Suppl. Fig. S4). The light-induced fluorescence changes were normalized to F_baseline_ immediately before the laser onset that remained consistent upon addition of the inhibitor DPO-1. The last 3 of 6 laser stimulation events paced at 0.5 Hz were averaged for analysis of DPO-1 inhibition. Only the stationary fluorescence values at the end of the ChR2(H134R) activation (“evaluation window” Suppl. Fig. S4) were used to determine the ∆F/F values

2 $${Normalized}\,{\rm{\Delta }}F=\frac{{\rm{\Delta }}{F}_{DPO}-{\rm{\Delta }}{F}_{bath}}{{\rm{\Delta }}{F}_{bath}}$$

With ΔF_DPO_ being the light-induced fluorescence change under DPO-1 application and ΔF_bath_ the light-induced fluorescence change in bath solution.

For optical dose-response experiments with DPO-1 we normalized the change in fluorescence (ΔF_*x*_) to the fluorescence change at maximal drug effect (∆F_max_):

3 $${\rm{\Delta }}F=\frac{{\rm{\Delta }}{F}_{x}-{\rm{\Delta }}{F}_{bath}}{{\rm{\Delta }}{F}_{max}-{\rm{\Delta }}{F}_{bath}}$$

### hNa_v_1.5 – QuasAr1

Light from the 593.5 nm laser (163 mW/mm^2^ at sample) for concomitant ArchT and QuasAr1 activation was collimated into a 910 μm diameter multimode optic fiber (Thorlabs) and fed into the fluorescent port of a Zeiss Axiovert 35 M inverted microscope. The 593.5 nm light was reflected by a dichroic mirror (650 nm) and focused on the back focal plane of a 40×/1.3NA Plan-Neofluoar oil objective (Zeiss, Germany). QuasAr1 fluorescence signals were filtered through a 665 nm LP filter and imaged at 3 kHz by a RedShirt NeuroCCD camera with 40 × 40 pixel resolution. 3–4 ROIs were offline manually selected on the soma of identified cells and the integrated raw fluorescence signal analyzed with Neuroplex software (RedShirtImaging, Fairfield, CT, USA) (Suppl. Fig. S2a). Only cells with a ∆F/F change ≥5% upon 473 nm light illumination were included into the analysis. The baseline fluorescence (F) was defined to a 50 ms window 200 ms after the start of the 593.5 nm laser illumination. The last three ∆F/F peaks triggered by 473 nm laser pulses (2 ms) at 5 Hz were averaged and the laser artifact removed from the signal by setting the averaged ∆F/F during 473 nm illuminations to 0 (Suppl. Fig. S2b). Non-specific response rundown between measurements when acquiring dose-response curves was corrected by automated exponential baseline subtraction in Clampfit:

4 $${\rm{Y}}=({y}_{0}-{y}_{plateau}){e}^{-Kx}+{y}_{plateau}$$

with *y*_0_ = 1, *y*_*plateau*_ = 0.8297, K = 0.5516 and x being the number of measurements within the series as observed under control conditions (Suppl. Fig. S3d). The fractional contribution of the ChR2 peak to the ∆F/F signal (ChR2_baseline_) was determined as the mean 473 nm light-induced peak ∆F/F signal under full lidocaine block (10 mM) and subtracted to isolate the hNa_v_1.5 induced ∆F/F value (ChR2_baseline_ with rundown compensation of x = 1).

### hERG - Di-4-ANBDQPQ

Cells were loaded with 20 μM Di-4-ANBDQPQ (kind gift from Leslie Loew, University of Connecticut)^27^ for 5 min before being placed into fresh bath solution for experiments. ChR2(L132C) was activated by 1 second long 473 nm light pulses (1 mW/mm^2^ at cells). The light from the 635 nm LED of the pE-4000 was passed through a 630/30 nm excitation filter and reflected by a 650 nm dichroic mirror (output at cells 5 mW/mm^2^). Emitted Di-4-ANBDQPQ fluorescence was passed through a 665 nm LP filter and imaged at 125 Hz by the RedShirt NeuroCCD camera with 80 × 80 pixels resolution. For each trace recorded, the field of view was moved to minimize the effect of local photodamage. The full field optical signal was spatially averaged to a single trace for analysis and inverted. The baseline fluorescence (F) was defined to a 300 ms time window 340 ms after imaging light onset. Bleaching was corrected by automated exponential baseline subtraction in Clampfit in each trace. For the quantification of drug action on hERG, the traces were aligned to the mean steady state fluorescence in a 1 second time window from 2.5 to 3.5 seconds after the offset of the 473 nm laser (Fig. 4a). Channel activity was quantified by the negative peak value of the ∆F/F signal in a time window of 1 s after the 473 nm light offset (Fig. 4a).

### hERG– BeRST1

Cells were loaded with 1 μM BeRST1^28^ (kind gift from Evan Miller, University of California) for 10 min. hERG experiments with the dye BeRST1 were performed with identical light sources, filters and settings as for Di-4-ANBDQPQ except that the 635 nm LED was increased to 20 mW/mm^2^ and the imaging frame rate was reduced to 40 Hz. Dose-response quantification was performed as described for Di-4-ANBDQPQ (Fig. 4a).

### General data analysis

All dose-response curves were fitted with a Hill function and IC_50_ ± standard error (s.e.) calculated in GraphPad prism:

5 $$y=\frac{1}{1+{10}^{(logIC50-X)\ast Hillslope)}}$$

Z-factors^55^ (Z’) were calculated with:

6 $$Z\text{'}=\frac{3\ast (SD\,of\,sample-SD\,of\,control)}{|mean\,of\,sample-mean\,of\,control|}$$

All data are indicated as mean ± standard deviation (s.d.) in the main text and as mean ± standard error of the mean (s.e.m.) in figures if not stated otherwise. Offline analysis was performed in Excel, Clampfit, OriginLab and GraphPad Prism. P-values in figures are indicated as follows: *p ≤ 0.05, **p ≤ 0.01, ***p ≤ 0.001, ****p ≤ 0.0001.

## Electronic supplementary material

Supplementary Information
