# Supplementary material for: Dynamic all-optical drug screening on cardiac voltage-gated ion channels
Source: Sci Rep. 2018 Jan 18;8:1153. doi: 10.1038/s41598-018-19412-z (PMC5773578; doi:10.1038/s41598-018-19412-z)
Supplement: Supplementary file 1 — Supplementary Information [file 41598_2018_19412_MOESM1_ESM.pdf]

Supplementary Information

Dynamic all-optical drug screening on cardiac voltage-gated ion channels

Jonas Streit[1] and Sonja Kleinlogel[1]*

[1]Institute of Physiology, University of Bern, Bühlplatz 5, 3012 Bern, Switzerland

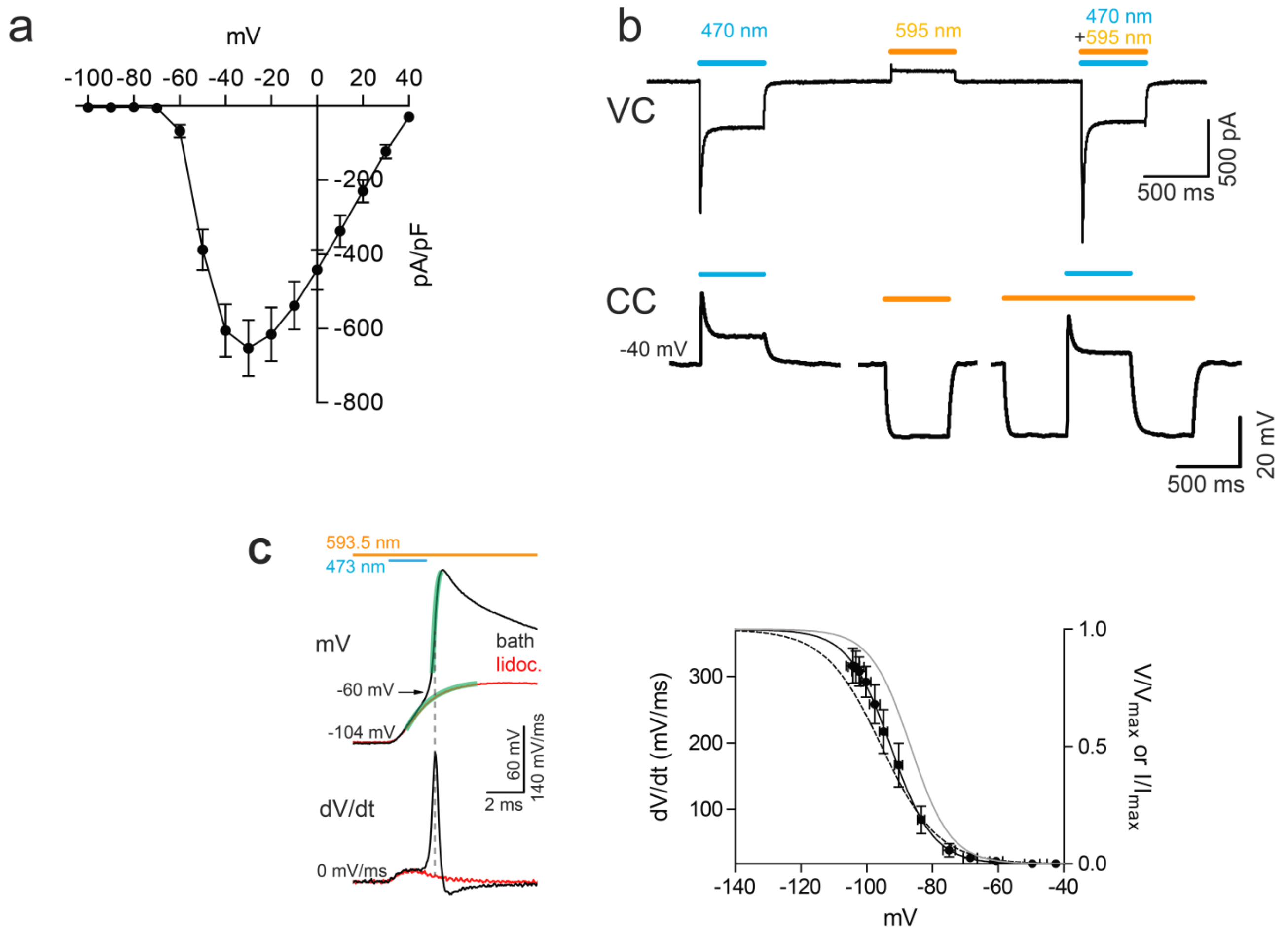

Supplementary Figure S1: Electrophysiological characterization of NG108-15 cells transiently co-expressing ChR2, ArchT, and $hNa_v1.5$. (**a**) $hNa_v1.5$ current-voltage relationship (protocol as shown in Suppl. Fig. S6, maximal current density: -652 ± 301 pA/pF, N=16). (**b**) Example of light-activated photocurrents (top trace, $V_{holding}$ -40 mV). Note that the yellow light co-illumination fully restores the ChR2 peak photocurrent at the second blue illumination. Light-induced membrane potential changes in the same cell are shown below. (**c**) Top left: Light-induced and $hNa_v1.5$-mediated cell depolarization, which was fully blocked by 10 mM lidocaine, revealing the underlying ChR2-mediated depolarization (red trace) and that clearly surpassed the $hNa_v1.5$ activation threshold (arrow). τ_{ON} fitting regions are shown in green. Left bottom: the onset of the fast sodium current was evident in the derivative (dV/dt) signal. Right panel: The fitted $hNa_v1.5$ steady state inactivation derived from either current recordings in VC (dashed line, according to Fig. 1a) or 595 nm light-tuning experiments quantifying either the maximal depolarization (Vmax , gray curve according to Fig. 1d panel 3) or dV/dt. Note that the dV/dt curve matches the VC data more closely than the Vmax curve. See also supplementary note 1 for further discussion.

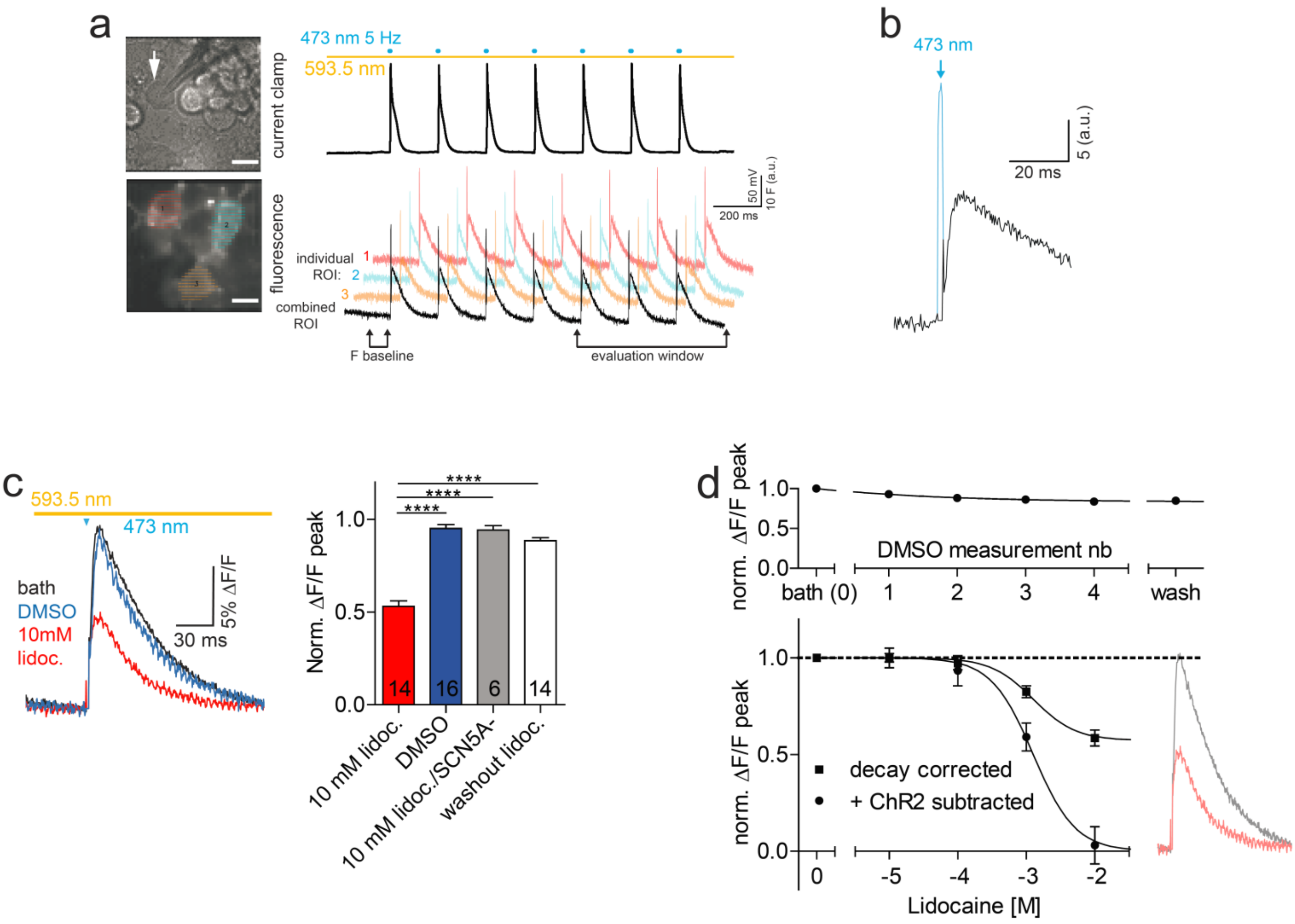

Supplementary Figure S2: Data processing for hNa$_{v}$1.5-LiEp. (**a**) Top: Current-clamp (2 ms blue light pulses during continuous yellow illumination). Bottom: LiEp QuasAr1 fluorescence traces from three single cells (ROI 1-3, color) and combined ROIs (black). The last 3 of 7 hNa$_{v}$1.5 spikes were averaged for quantification, marked as "evaluation window". Scale bar in the photomicrographs is 20 µm. (**b**) 473 nm light stimulation caused a "laser artifact" (blue) in the fluorescence readout. This laser artifact was removed from the combined ROI signal offline. (**c**) Lidocaine block monitored by LiEp. Application of saturating lidocaine concentrations (10 mM) fully abolished the hNa$_{v}$1.5 activity, isolating the residual ChR2 peak (red trace), whereas application of the solvent DMSO alone had no effect (Plot: quantification). Application of lidocaine in hNa$_{v}$1.5-lacking cells (SCN5A-) did not reduce the signal (all values normalized to paired recording in bath solution). N for each sample is given within the columns. (**d**) Top: QuasAr1 signal rundown during repetitive imaging (N=11, each measurement with 7 stimulation pulses and quantified as shown in panel (a)) was fit with a single exponential decay function and used for LiEp data offline correction. DMSO concentration at respective measurement number were: (1) 0.01‰, (2) 0.1‰, (3) 1‰, (4) 1%. Further, the fractional ChR2 peak component (from (c)) was used for max-min scaling (shown at bottom).

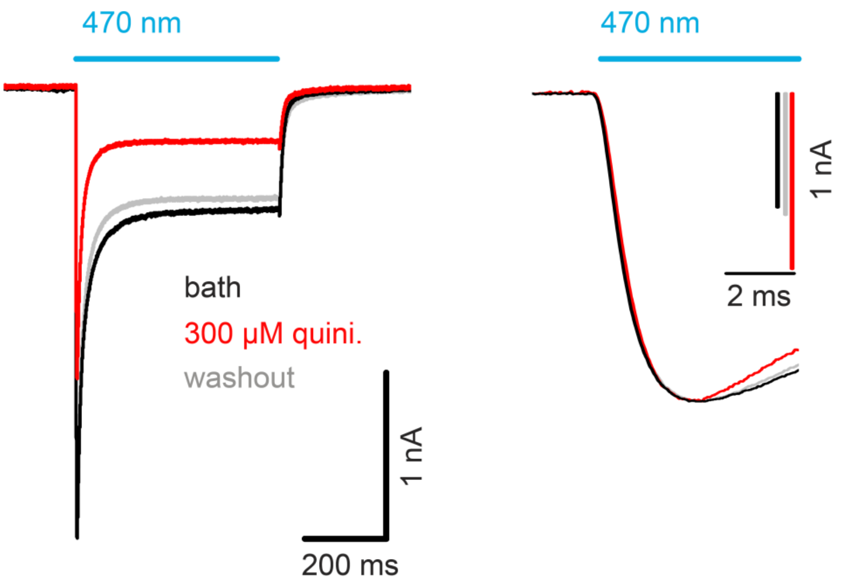

Supplementary Figure S3: 473 nm light-triggered ChR2 photocurrents under maximally applied concentrations of quinidine (300 µM). The photocurrent onset is temporally enlarged on the right (currents scale normalized to peak current). Both ChR2 peak and stationary photocurrents were reduced. The ChR2 current onset kinetics, which is detrimental to reach the $hNa_v1.5$ threshold, remained unaffected.

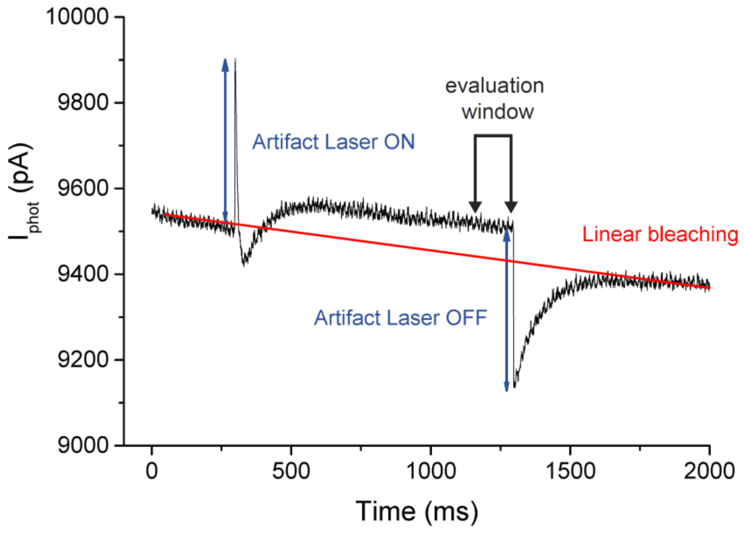

Supplementary Figure S4: Linear bleaching correction, laser artifact subtraction and sign inversion of the $hK_v1.5$ LiEp raw data detected with the fluorescent probe RH421. Examples for corrected traces are shown in Fig. 6d. Fluorescence was quantified from the region marked as “evaluation window”.

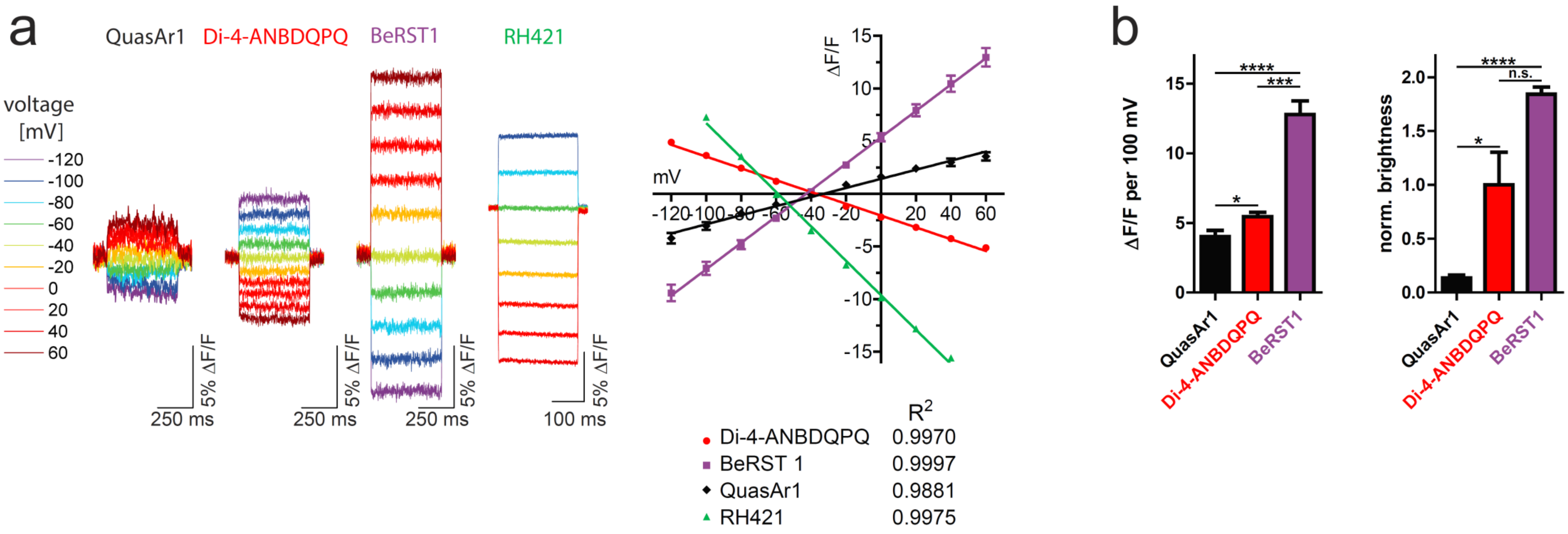

Supplementary Figure S5: Characterization of the used voltage sensors. (**a**) Left: single-cell optical ΔF/F responses recorded from patched cells subjected to 500 ms voltage steps. Right: All indicators delivered linear voltage readouts throughout the LiEp signal range (R^2 values for linear curve fits shown below). (**b**) Quantification of the ΔF/F signal amplitudes and the normalized dye brightness relative to Di-4-ANBDQPQ. QuasAr1 N=5, Di-4-ANBDQPQ N=4, BeRST1 N=3, RH421 N=1 for (a) and (b). For data and exact p values in (b), see supplementary table S2.

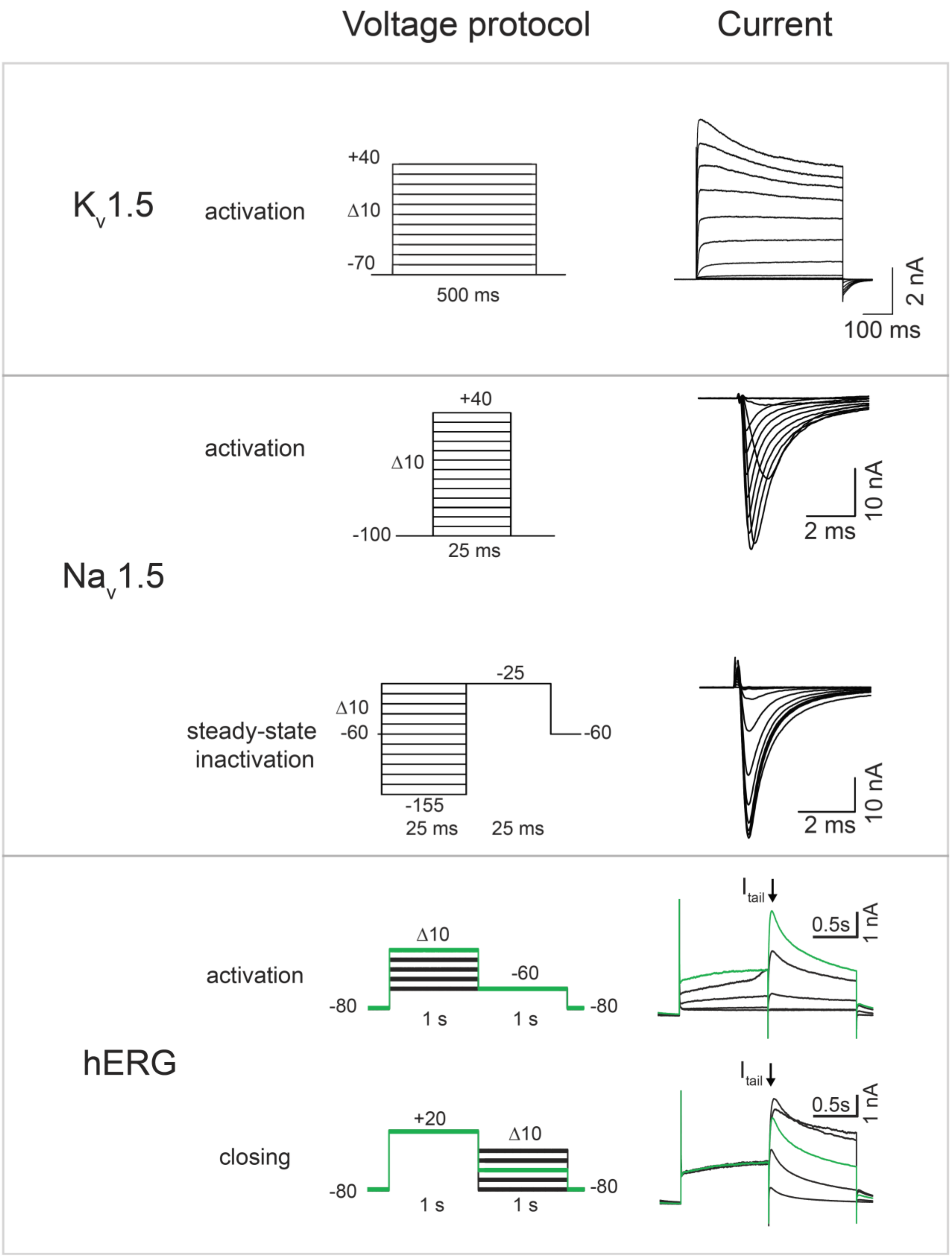

Supplementary Figure S6: Voltage-clamp step protocols. $hK_v1.5$ currents were activated by a series of 500-ms pulses from -70 mV to +40 mV in 10 mV intervals from a holding potential of -70 mV. $hNa_v1.5$ currents were elicited from a holding potential of -100 mV with 25 ms steps ranging from -100 mV to +40 mV with 10 mV increments and steady-state inactivation was examined with 25 ms step ranging from -155 mV to -25 mV with 10 mV increments followed by a 25 ms step to 25 mV. hERG currents were elicited by a 1 s step to +20 mV (+50 mV for drug experiments) followed by a 1 s step to -50 mV before returning to the holding potential of -80 mV.

	**DMSO control**	**Flecainide 500 µM**	**Quinidine‡ 300 µM**	**Lidocaine‡ 10 mM**
ChR2 I_{peak} dark-adapted	99.4 ± 12.2%	86.7 ± 7.8% p=0.113	56.8 ± 7.0% p<0.0001	87.1 ± 9.5% p=0.016
N	13	3	5	10

	**DMSO control**	**Quinidine 100 µM**	**Terfenadine 10 µM**	**Astemizole 30 µM**
CatCh $I_{stationary}$	94.8. ± 2.4%	91.7 ± 9.9% p=0.51	93.7 ± 2.6% p=0.600	83.1 ± 11.0% p=0.147
N	3	4	3	3

Supplementary table S1: Effect of saturating concentrations of the tested drugs on the ChR2 peak and CatCh stationary photocurrents (remaining photocurrent given in %). ‡The ChR2 activity still sufficed to surpass the $hNa_v1.5$ activation threshold (as shown for 10 mM lidocaine in Suppl. Fig. S1c, see also Fig. 1d panel 2). Also compare with Suppl. Fig. S3.

Published data							LiEp				
**Voltage reporter** (with Ref.)	**peak λ_{abs}** (nm)	**peak λ_{em}** (nm)	**ill. intensity** (mW/mm^2)	**$\Delta F/F$ per 100** mV	**tau (on)**	**photo-bleaching half-life**	**λ_{ex}** (nm)	**ill. Intensity** (mW/mm^2)	**$\Delta F/F$ per 100 mV p-value**	**brightness relative to Di-4-ANBDQPQ p-value**	**N**
**QuasAr1**[1]	590	715	3000	33%	0.053 ms	5 min	595	163	4.0±1.0%	0.13±0.06	5
**Di-4-ANBDQPQ**[2]	603	800-850	1	10-20%	<<ΔmV	N/A	635	5	5.4±0.6% $p_{QuasAr1} = 0.0416$	1.0±0.6 $p_{QuasAr1} = 0.0145$	4
**BeRST1**[3]	658	683	60-1600	24%	<<ΔmV	5 min	635	20	12.7±1.7% $p_{QuasAr1} < 0.0001$ $p_{Di\text{-}4\text{-}ANBDQPQ} = 0.0004$	1.8±0.1 $p_{QuasAr1} < 0.0001$ $p_{Di\text{-}4\text{-}ANBDQPQ} = 0.0682$	3
**RH421**[4,5]	509	719	N/A	21%	<<ΔmV	N/A	565	<0.01	17%	N/A	1

Supplementary table S2: Overview of the properties of the used voltage sensors (see also Suppl. Fig. S5). Values in table as mean ± s.d. where applicable, p values in comparison with respective other dyes are shown for LiEp. RH421 imaging was performed with a different microscope and detector and obtained values were therefore not directly comparable to other dyes.

Supplementary note 1

Considerations for $hNa_v1.5$ activity quantification with LiEp.

For optical ion channel quantification with a fluorescent potential sensitive probe one needs to consider that changes in transmembrane voltage result in a non-linear relationship between channel conductance and channel current. For this reason, the rise time (dV/dt) of the $hNa_v1.5$ action potential (AP) might be a more accurate estimate of the $hNa_v1.5$ conductance than the AP amplitude. However, in all-optical $hNa_v1.5$ experiments with QuasAr1 (Fig. 2, Suppl. Fig 2) the low sampling rate (3kHz), low signal-to-noise ratio compared to CC and the presence of an optical artefact during blue laser stimulation (Suppl. Fig. 2b) was detrimental for the analysis of AP onset kinetics in optical data. In addition, rise to peak time was significantly lower for QuasAr1 than in membrane potential recordings in CC (rise time 10% to 90% AP peak height starting from 473 nm light onset: CC 2.01 ± 0.38 ms, N=3; QuasAr1 ∆F/F readout 6.35 ± 1.81 ms, N=30; p=0.0003), resulting in insufficient temporal precision to analyse AP upstroke velocities. However, the AP amplitude was previously successfully used to estimate IC_{50} values of hNa_v antagonists[6,7].

The slow QuasAr1 onset kinetics stands in contrast to the original report (τ_{ON} = 0.053 ms)[1], which used much higher light intensities (see Suppl. Table 2) that we did not exploit due to cellular phototoxicity of such bright light. A consequence of using the signal amplitude for optical IC_{50} quantifications, however, is a small but systematic discrepancy of drug IC_{50} values determined by LiEp compared to those determined by VC (LiEp approx. 3-5 fold less sensitive than VC). False negative results with LiEp will be avoided once more light-sensitive and spectrally red-shifted voltage-sensors are available or, when using QuasArs, by either reducing the hit threshold or increasing the tested drug concentrations 3-5 fold.

Supplementary References

1 Hochbaum, D. R. *et al.* All-optical electrophysiology in mammalian neurons using engineered microbial rhodopsins. *Nature methods* **11**, 825-833, doi:10.1038/nmeth.3000 (2014).

2 Matiukas, A. *et al.* Near-infrared voltage-sensitive fluorescent dyes optimized for optical mapping in blood-perfused myocardium. *Heart rhythm : the official journal of the Heart Rhythm Society* **4**, 1441-1451, doi:10.1016/j.hrthm.2007.07.012 (2007).

3 Huang, Y. L., Walker, A. S. & Miller, E. W. A Photostable Silicon Rhodamine Platform for Optical Voltage Sensing. *Journal of the American Chemical Society* **137**, 10767-10776, doi:10.1021/jacs.5b06644 (2015).

4 Grinvald, A., Fine, A., Farber, I. C. & Hildesheim, R. Fluorescence monitoring of electrical responses from small neurons and their processes. *Biophysical journal* **42**, 195-198, doi:10.1016/s0006-3495(83)84386-0 (1983).

5 Matson, M., Carlsson, N., Beke-Somfai, T. & Norden, B. Spectral properties and orientation of voltage-sensitive dyes in lipid membranes. *Langmuir : the ACS journal of surfaces and colloids* **28**, 10808-10817, doi:10.1021/la301726w (2012).

6 Huang, C. J. *et al.* Characterization of voltage-gated sodium-channel blockers by electrical stimulation and fluorescence detection of membrane potential. *Nature Biotechnology* **24**, 439-446, doi:10.1038/nbt1194 (2006).

7 Zhang, H., Reichert, E. & Cohen, A. E. Optical electrophysiology for probing function and pharmacology of voltage-gated ion channels. *eLife* **5**, doi:10.7554/eLife.15202 (2016).
